# Thyroglobulin levels before radioactive iodine therapy and dynamic risk stratification after 1 year in patients with differentiated thyroid cancer

**DOI:** 10.1590/2359-3997000000308

**Published:** 2017-12-01

**Authors:** Leonardo Bandeira, Rosália do Prado Padovani, Ana Luiza Ticly, Adriano Namo Cury, Nilza Maria Scalissi, Marília Martins Silveira Marone, Carolina Ferraz

**Affiliations:** 1 Irmandade da Santa Casa de Misericórdia de São Paulo Departamento de Medicina Serviço de Endocrinología São Paulo SP Brasil Serviço de Endocrinología, Departamento de Medicina, Irmandade da Santa Casa de Misericórdia de São Paulo (ISCMSP), São Paulo, SP Brasil; 2 Irmandade da Santa Casa de Misericórdia de São Paulo Serviços de Medicina Nuclear São Paulo SP Brasil Serviços de Medicina Nuclear, Irmandade da Santa Casa de Misericórdia de São Paulo (ISCMSP), São Paulo, SP Brasil

**Keywords:** Dynamic risk stratification, radioactive iodine therapy, thyroglobulin, thyroid cancer

## Abstract

**Objectives:**

We sought to assess the relationship between stimulated thyroglobulin (sTg) before radioactive iodine therapy (RIT), and the dynamic risk stratification 1 year after treatment, and to establish the utility of the sTg as a predictor of response to therapy in these patients. A retrospective chart review of patients with differentiated thyroid cancer (DTC) who underwent RIT after surgery and were followed for at least 1 year, was carried out.

**Subjects and methods:**

Patients were classified according to the dynamic risk stratification 1 year after initial treatment. The sTg values before RIT were compared among the groups. ROC curve analysis was performed.

**Results:**

Fifty-six patients were enrolled (mean age 44.7 ± 14.4 years, 80.7% had papillary carcinoma). Patients with excellent response had sTg = 2.1 ± 3.3 ng/mL, those with indeterminate response had sTg = 8.2 ± 9.2 ng/mL and those with incomplete response had sTg = 22.4 ± 28.3 ng/mL before RIT (p = 0.01). There was a difference in sTg between excellent and incomplete response groups (p = 0.009) while no difference was found between indeterminate and either excellent or incomplete groups. The ROC curve showed an area under the curve of 0.779 assuming a sTg value of 3.75 ng/mL.

**Conclusion:**

Our study results suggest that the higher the sTg before RIT, the greater the likelihood of an incomplete response to initial treatment. A sTg cut-off of 3.75 ng/mL was found to be a good predictor of response to initial treatment in patients with DTC.

## INTRODUCTION

Differentiated thyroid carcinomas (DTC) originating from follicular cells account for over 90% of thyroid cancers. DTC comprise both papillary and follicular carcinomas, with the former type being the most common type (around 80% of DTC). The incidence of DTC has increased in recent decades and is 2-4 times more frequent in women than in men, peaking at 40-50 years of age. This disparity between genders narrows progressively with age, with rates almost equal in older adults (1,2).

Based on global consensus on management of DTC, initial treatment includes thyroidectomy with or without lymph node chain resection, in association with administration of I^131^ radioactive iodine therapy (RIT) in patients with significant risk of death or recurrence (3-5). After initial therapy, the daily administration of suppression doses of levothyroxine has an important adjuvant role in high-risk patients since suppression of thyroid stimulating hormone (TSH) inhibits tumor growth and progression, thereby reducing the risk of disease recurrence and associated death (6-9).

Ideally, initial staging of DTC should be carried out shortly after surgery. Several classifications have been developed for staging. Systems for classifying death risk include the MACIS and TNM, with the latter being the most widely used. More recently, new classification systems have been created for assessing risk of disease recurrence and persistence, for example the American Thyroid Association (ATA) risk stratification (4,10).

With the aim of complementing initial staging and assessing response to treatment, a new classification that restages patients 1 year after initial therapy was recently developed (dynamic risk stratification using response to therapy restaging system). Using Tg and anti-Tg antibodies levels, USG and other imaging methods as parameters, patients are reclassified as having excellent, indeterminate (acceptable), or incomplete (biochemical or structural) response to treatment, which then modifies subsequent therapy and follow-up (4,11). Under this classification, excellent response is defined as the presence of stimulated Tg (sTg) < 1 ng/mL (with absent anti-Tg levels) and negative imaging scans. The presence of elevated Tg (suppressed ≥ 1 ng/mL or stimulated ≥ 10 ng/mL) or rising anti-Tg levels defines incomplete response, which is either structural (local/ regional or distal disease evident on imaging exams) or biochemical (no disease evident). Indeterminate response is established in the presence of non-specific findings on cervical ultrasound, suppressed Tg < 1 ng/ mL and sTg < 10 ng/mL or stable/declining anti-Tg levels (Table 1). According to this classification, in the event of excellent response to treatment, the frequency and intensity of monitoring can be reduced and TSH target raised (0.5-2 mUI/L if initial ATA low or intermediate risk patients; 0.1-0.5 mUI/L if initial ATA high risk patients). Patients attaining an indeterminate response should be kept under approximately the same TSH target as the excellent response and undergo more frequent follow-up, for later reclassification. If biochemical or structural response, patients should be kept under suppression (TSH < 0.1 mUI/L for structural incomplete response; TSH = 0.1-0.5 mUI/L for biochemical incomplete response). In case of elevated Tg, investigation by imaging studies and more aggressive therapeutic are recommended (4,11).

**Table 1:** Dynamic risk stratification (restratification)

Excellent response	Indeterminate response	Incomplete response
– Negative imaging – Suppressed Tg < 0.2 ng/mL or stimulated Tg < 1.0 ng/mL – Absent anti-Tg levels	– Non-specific findings on imaging studies – Faint uptake in thyroid bed on RAI scanning – Suppressed Tg detectable but < 1 ng/mL – Stimulated Tg detectable but < 10 ng/mL or stable or declining anti-Tg levels	– Suppressed Tg ≥ 1 ng/mL or stimulated Tg ≥ 10 ng/mL or rising anti-Tg levels – Biochemical, if negative imaging – Structural, if evidence of disease on imaging studies

Adapted from Haugen and cols. (4) and Tuttle and Leboeuf (11). Tg: thyroglobulin.

Serum Tg is the primary tumor marker used in follow-up of patients with DTC for detecting the disease after initial treatment (12). In recent years, the utility of Tg measured immediately before ablative therapy with I^131^ and after surgery (Tg before RIT) as a prognostic marker of disease progression has been confirmed (13-17). No previous studies, however, have sought to establish the relationship between Tg before RIT and the new dynamic risk stratification.

In order to better define the role of Tg before RIT as a prognostic factor, the objectives of the present study were: 1) To determine whether a relationship exists between sTg levels (TSH > 30 mUI/L) before RIT and after thyroidectomy, and the dynamic risk stratification at 1 year after therapy in patients with DTC; 2) To determine a possible cut-off for sTg before RIT and after thyroidectomy, as a predictor of prognosis.

## SUBJECTS AND METHODS

A retrospective study was conducted analyzing the relationship between sTg levels before RIT and the dynamic risk stratification at 1 year after initial therapy in patients with DTC who undergone thyroidectomy. Data were collected from medical charts of patients referred for RIT after thyroidectomy at the Laboratory of Nuclear Medicine of the Santa Casa Hospital of Sao Paulo.

Sixty patients were eligible for the study. The definitive diagnosis of DTC was reached based on the results of pathological examination of the surgical specimen. The study included all patients diagnosed with DTC of any histological subtype submitted to initial surgical treatment (thyroidectomy) followed by RIT. The I^131^ activity was administered after preparing the patient with a discontinuation of thyroid hormone and an iodine-poor diet as American Thyroid Association (ATA) recommendations (4). The exclusion criteria were patients who had partial thyroidectomy and those with positive anti-Tg antibodies.

Data were collected for age, surgery type, histological type of carcinoma, initial staging by TNM and ATA classifications, I^131^ (RIT) activity administered, sTg level before RIT, and response to treatment at 1 year after RIT based on the dynamic risk stratification (4,11). Tg and anti-Tg antibody analyses were performed at the same laboratory for all patients using the same assay (chemiluminescent Immulite 2000, Siemens).

Statistical analyses were carried out using the statistics package SPSS version 13.0. The level of statistical significance adopted was p ≤ 0.05. Absolute (n) and relative (%) frequencies were analyzed for qualitative variables, while decimal measures (mean, standard deviation, standard error and median) were calculated for quantitative variables.

Patients were divided into 3 groups according to the dynamic risk stratification (excellent, indeterminate or incomplete response) at 1 year after initial treatment (4,11). Although it is known that patients with biochemical incomplete response do have better outcomes than patients with structural incomplete response (4), those from both groups were pooled into a single incomplete response group given they need similar treatment (suppression levothyroxine therapy and more closer follow-up). The Kruskal-Wallis nonparametric test was performed to compare sTg levels before RIT and after thyroidectomy among the 3 groups. The Mann-Whitney non-parametric test was used for multiple comparisons between the specific groups. A ROC curve was built to define a cut-off value for sTg before RIT for predicting response to initial treatment after 1 year.

## RESULTS

Sixty patients were initially included in the study. Four patients tested positive for anti-Tg antibodies and were subsequently excluded. All participants were submitted to total thyroidectomy (TT) or totalization after partial thyroidectomy. In these cases, I^131^ dose and sTg measurement before RIT were performed after totalization and therefore these patients were not excluded. Among the 56 patients enrolled in the study (supplemental Table 1), 46 (80.7%) had papillary carcinoma while the remainder had follicular carcinoma. Among patients with papillary carcinoma, 70% had the classic variant subtype, 26% the follicular variant and 4% had other more aggressive variants. Among the cases with follicular carcinoma, 30% had the Hürthle cell variant. Participant age had a range of 20-76 years, mean of 44.7 ± 14.4 years and median of 47 years.

According to TNM staging (4), 51.8% of patients were classified as stage I, 3.6% stage II, 28.6% stage III and 16.1% stage IV. With regard to risk of disease recurrence/persistence by the ATA classification, 14.3% of patients had low risk, 69.6% intermediate risk and 16.1% high risk of recurrence. The I^131^ dose administered ranged from 100 to 250 mCi, with a mean of 184.1 ± 55.8 and median of 200 mCi.

sTg value after TT and before RIT ranged from 0.5 to 81 ng/mL, with mean of 6.4 ± 13.8 and median of 0.8 ng/mL. When patients were restaged, 67.3% had an excellent response to treatment, 15.4% indeterminate and 17.3% incomplete response 1 year after initial therapy. From our initial stated ATA low risk patients, 87.5% had an excellent response to the proposed treatment while 12.5% evolved with incomplete response. Among the intermediate risk group patients 61.1% had an excellent response, 22.2% an indeterminate response and 16.7% evolved with incomplete response. Finally, the high risk group showed an excellent response in 75% of the patients while in 25% there was an incomplete response (Table 2).

**Table 2:** Initial ATA recurrence risk and the dynamic risk stratification after 1 year

		Dynamic risk stratification			
		Excellent response	Indeterminate response	Incomplete response	Total
ATA Recurrence Risk	Low	87.5%	0%	12.5%	100%
	Intermediate	61.1%	22.2%	16.7%	100%
	High	75.0%	0%	25.0%	100%
	Total	67.3%	15.4%	17.3%	100%

**Figure 1:**
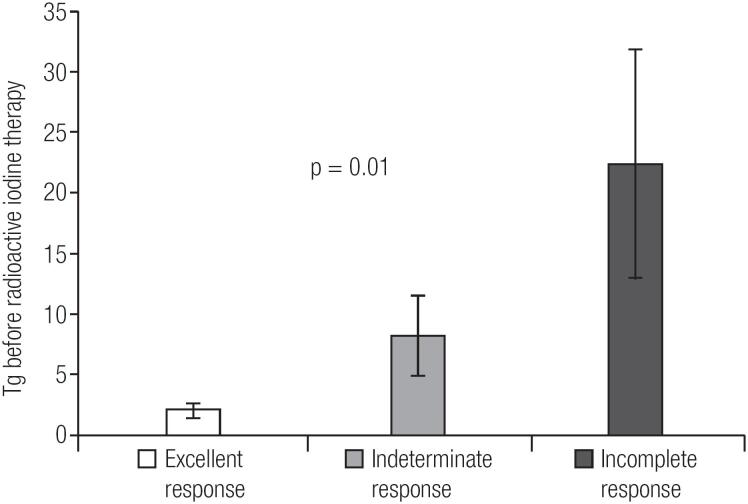
Stimulated Tg before radioactive iodine therapy (mean ± SE) and dynamic risk stratification 1 year after initial treatment. Difference in Tg values among the 3 groups of dynamic risk stratification (p = 0.01).

Table 3 shows the baseline characteristics of the dynamic risk stratification groups. Patients showing an excellent response to treatment after 1 year had a mean sTg before RIT of 2.1 ± 3.3 and median of 0.7 ng/mL; those with indeterminate response had a mean sTg before RIT of 8.2 ± 9.2 and median of 4.6 ng/mL; whereas patients with incomplete response had a mean sTg before RIT of 22.4±28.3 and median of 6.3 ng/mL (p = 0.01, Figure 1).

**Table 3:** Baseline characteristics of the patients ± SD

		Excellent response (n = 37)	Indeterminate response (n = 9)	Incomplete response (n = 10)	p value
Age (years)		46.4 ± 14.7	38.2 ± 13.2	42.2 ± 14.3	0.443
Type of carcinoma					
	Papillary, n (%)	31 (83.8)	8 (88.9)	7 (70.0)	0.509
	Follicular, n (%)	6 (16.2)	1 (11.1)	3 (30.0)	
TNM Staging					
	I, n (%)	20 (54.1)	5 (55.6)	5 (50.0)	
	II, n (%)	1 (2.7)	0 (0.0)	0 (0.0)	0.979
	III, n (%)	11 (29.7)	2 (22.2)	3 (30.0)	
	IV, n (%)	5 (13.5)	2 (22.2)	2 (20.0)	
ATA Classification					
	Low risk, n (%)	7 (18.9)	0 (0.0)	1 (10.0)	0,194
	Intermediate risk, n (%)	24 (64.9)	9 (100.0)	6 (60.0)	
	High risk, n (%)	6 (16.2)	0 (0.0)	3 (30.0)	
sTg after TT and before RIT (ng/mL)		2.1 ± 3.3	8.2 ± 9.2	22.4 ± 28.3	0.01
RIT dose (mCi)		177.0 ± 57.2	195.7 ± 53	210.0 ± 39.4	0.111

sTg: stimulated thyroglobulin; TT: total thyroidectomy; RIT: radioactive iodine therapy.

**Figure 2:**
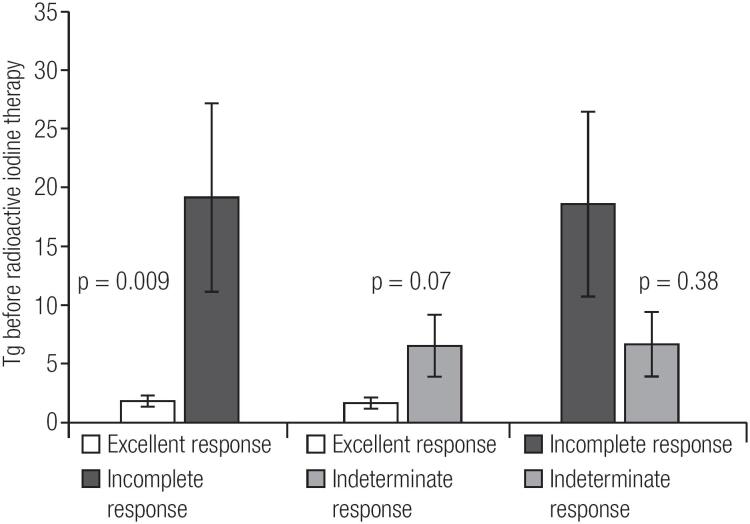
Stimulated Tg before radioactive iodine therapy (mean and SE) and comparison among groups of dynamic risk stratification 1 year after initial treatment. Difference in Tg between excellent and incomplete response groups (p = 0.009). Comparisons between indeterminate and excellent response groups (p = 0.072) and between indeterminate and incomplete response groups (p = 0.385) were not statistically significant.

**Figure 3:**
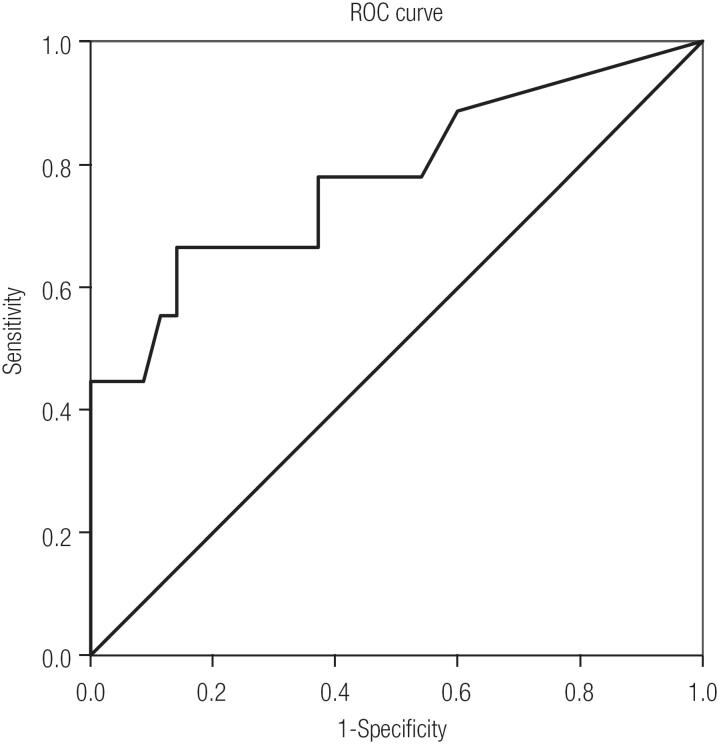
ROC curve assuming a Tg value of 3.75 ng/mL before RIT. Area under the curve = 0.779.

Comparison of restaging groups revealed a difference in sTg values before RIT between the excellent and incomplete response groups (p = 0.009). Comparisons between sTg values in the indeterminate and the excellent response groups (p = 0.072) and between the indeterminate and incomplete response groups (p = 0.385) were not statistically different (Figure 2). If patients with sTg value before RIT < 1 ng/mL are excluded from the analysis (in order to avoid pulling down the Tg values), the difference in sTg before RIT between the excellent and incomplete response groups maintains significant (p = 0.007). Again, between the excellent and indeterminate response groups (p = 0.76) and between the indeterminate and incomplete response groups (p = 0.273) there were no significant difference between sTg values. For sTg values measured before RIT, a cut-off of 3.75 ng/mL had a sensitivity for predicting poor response to treatment of 66.7% while the specificity was 85.7%. Analysis of the ROC curve showed an area under the curve of 0.779 (Figure 3).

## DISCUSSION

The prevalence of DTC has risen in recent years, largely due to the increase in diagnosis of microcarcinomas (tumors up to 1 cm across at widest point) and representing the fourth most prevalent malignant neoplasm in Brazilian women (5,18-20). Studies suggest that the majority of DTC does not clinically progress, explaining the continued low death rates despite increased incidence (21,22).

Although most patients with DTC have a good outcome with conventional therapy, a considerable percentage of them has unfavorable response (21). Thus, it is important to distinguish between patients requiring more aggressive treatment from those that can be spared unnecessary treatment and procedures. Consequently, it is essential to perform initial staging of the disease (5). Several systems have been developed to assess prognosis, risk of recurrence and death, to help inform decisions on post-operative therapy and the frequency and intensity of follow-up, as well as to standardize language and facilitate communication of the multi-disciplinary team involved in the follow-up of these patients (3-5). However, these classification systems are, in general, only static representations of the patient after surgery and are not modifiable during follow-up, proving useful only to guide initial therapeutic measures. In an effort to optimize the follow-up of this patient group, a dynamic risk stratification/restaging was recently developed (11). This system has been incorporated into the new ATA guidelines on the management of DTC (4).

sTg (in the presence of TSH > 30 mUI/l) is the best means of detecting remnant thyroid tissue after initial treatment in patients with DTC. Elevated TSH, whether endogenous or recombinant, stimulates the uptake of iodine and promotes release of more Tg from thyroid remnants and metastatic lesions (23), thereby improving the accuracy of the scan. However, despite its importance as a biochemical marker for monitoring DTC, serum Tg is not classically used as an initial prognostic value.

Guidelines for DTC management indicate the use of sTg levels at between 6 and 12 months after initial treatment for the diagnosis of disease persistence and/ or recurrence (3,4,24). Thus, this diagnosis is often delayed by up to 1 year. Although the use of sTg before RIT and after surgery was initially questioned, because the remnant healthy thyroid tissue also contributes to its production, a number of studies have shown the prognostic value of sera sTg measured at this time point (13-17). The marker can be an early indicator of patient response, allowing treatment to be started immediately in the event of suspected unfavorable outcome.

Heemstra and cols. assessed the prognostic value of sTg at different time points and concluded that sTg before RIT was an independent prognostic marker of remission, while Tg measured after initial therapy (at 6 months, 2 years and 5 years) had utility for predicting death due to the disease (15). Studies found that sTg before RIT predicts the presence of metastases and empirically suggests the administration of high doses of I^131^ if this marker is elevated (13,16). Another two studies showed the higher the values of sTg before RIT, the greater the risk of disease persistence/recurrence (14,17).

We report a retrospective study analyzing the relationship between stimulated Tg levels after thyroidectomy and before RIT, and dynamic risk stratification 1 year after therapy in patients with DTC.

Around 80% of patients had papillary carcinoma while the remainder had follicular carcinoma, a similar rate to that found in the literature (1).

Most patients had an excellent response (67.3%) according to restaging, an expected outcome given that the majority of patients with DTC has a favorable response after initial therapy (21). A considerable number of patients, however, had an indeterminate or incomplete response, indicating the need for closer follow-up and more aggressive therapeutic measures in these groups.

In the present study, a significant difference in sTg levels before RIT was found among the restaging groups 1 year after therapy. The excellent response group had lower levels, the indeterminate group had intermediate levels of sTg and the incomplete group had higher levels (2.1 ± 3.3 vs. 8.2 ± 9.2 vs. 22.4 ± 28.3 ng/mL, p = 0.01). A statistically difference was detected between the excellent and incomplete groups (p = 0.009). Comparisons involving patients with indeterminate response revealed no statistical significance, results that might be explained by the small number of patients (n = 8) included in this group (p = 0.072 vs. excellent response group; p = 0.385 vs. incomplete response group).

Thus, the higher the sTg value before RIT, the greater the likelihood of the patient having an incomplete, or even an indeterminate response to treatment 1 year after initial therapy.

Analysis of the ROC curve showed good accuracy using a Tg value before RIT of 3.75 ng/mL (area under curve of 0.779), whereas ideally a diagnostic test should have an area under the curve > 0.7 to have at least moderate accuracy (25). In this analyzed cohort, another cut-off of Tg value before RIT comparing patients that evolve “better”, that means excellent, indeterminate and biochemical incomplete response, with patients that do not evolve well (structural incomplete response) can't be done due to the low number of patients with structural disease. Further patients must be added to the latest group in order to find a new cut-off to predict structural incomplete disease.

Studies assessing sTg before RIT as a prognostic value have shown different cut-off values for predicting better or worse outcomes. While Ronga and cols. (16) suggested administration of a high dose of I^131^, claiming a greater risk of metastasis, if the sTg value before RIT exceeds 69.7 ng/mL, other studies suggest a much lower cut-off, namely 5-10 ng/mL, in which greater levels would increase the risk of metastasis and also the rate of failed RIT ablation (26-28). Melo and cols. (17) established a cut-off of 7.2 ng/mL, in which levels of sTg before RIT lower than this had a high probability of remission after 1 year. Kim and cols. (14) suggested a lower cut-off point as an indicator of disease remission (negative predictive value of 98.4% for sTg before RIT ≤ 2 ng/mL), although their study excluded patients with metastasis. Hall and cols. (29) determined that a sTg level above 20 ng/mL is an independent predictor of disease recurrence. Other study found a greater cutoff (50 ng/mL) as a predictor of disease persistence/ recurrence (30), but this study enrolled only high-risk patients.

Despite the disparity in cut-off values before RIT, most of the related medical literature sees a sTg value ≥ 10 ng/mL as a predictor of negative response to initial treatment (13,31-36). In our study, we found that a Tg value before RIT ≥ 3.75 ng/mL had good specificity (85.7%) with acceptable sensitivity (66.7%) for predicting a not so good (incomplete or indeterminate) response to initial treatment.

Our results showed that sTg level before RIT can point out the response to initial therapy after 1 year. Therefore, as shown in other studies (13,14,16,17), it can be used to indicate prognostic. No previous studies, however, have attempted to associate sTg level before RIT with the new dynamic risk stratification adopted by the ATA.

This study has several limitations. Firstly, although RIT was administered at the same laboratory, using the same protocols and type of preparation, the surgery and follow-up of patients was not carried out at the same center. The laboratory of Nuclear Medicine of the Santa Casa hospital of Sao Paulo, as a referral center, receives patients from a number of other centers in the region specifically to undergo RIT. Consequently, variables such as surgical ability, extent of surgery (with or without lymphadenectomy) and follow-up protocols specific to each service may represent confounding factors. Secondly, the relationship between sTg before RIT and restaging was not tested in patients submitted to partial thyroidectomy or those not receiving RIT after TT, and results reported do not apply to such cases.

It is important to note that anti-Tg antibodies are associated with disease activity (4) and interfere the Tg assay (37). Accordingly, patients testing positive for antibodies were excluded. Of the original sample, 6.6% tested positive for anti-Tg, lower than the rate described in the literature (15-20%) (38,39). This disparity may have occurred because some patients were not referred for therapy straight away and, upon withdrawal of antigenic stimulus after surgery, anti-Tg levels steadily decline (40,41). Another exclusion criterion was for patients submitted to partial thyroidectomy because Tg values for assessing response are higher in these patients given that part of the thyroid remains (42). However, all patients included were submitted to total thyroidectomy or totalization after partial thyroidectomy.

In this study, it was concluded that sTg before RIT is associated with dynamic risk stratification (restaging) at 1 year after therapy in patients with DTC. Higher Tg levels were found in patients that had indeterminate, and particularly incomplete, response. Thus, the higher the Tg level before RIT and after surgery, the greater the likelihood of having an incomplete response to initial treatment. Therefore, we suggest that sTg before RIT can serve as a predictor of response to initial treatment and that a value ≥ 3.75 ng/mL represents a good cut-off for incomplete response.

## SUPPLEMENTAL MATERIAL

**Supplemental table 1:** Summarized data from all patients.

Patients’ initials	Age	Histological type	TNM staging	ATA classification	sTg	Dynamic risk stratification
SNG	20	F	I	I	0,8	IndR
ICR	51	P	III	I	0,5	IndR
TCK	58	P	IV	I	0,5	IndR
FIVS	35	P	I	I	2	IndR
MRRL	48	P	IV	I	12	IndR
EJA	29	P	I	I	1,1	IndR
CH	27	P	I	I	8,1	IndR
FSS	28	P	I	I	18,9	IndR
EFLS	48	P	III	I	24	IndR
MSFA	48	F	III	I	0,8	ER
RFZ	32	F	I	I	0,5	ER
JDJR	52	F	IV	H	0,7	ER
MGCS	29	F	IV	H	2,7	ER
LSV	25	F	I	I	1,3	ER
MAPS	50	F	II	L	2,8	ER
SNR	52	P	III	I	0,5	ER
AVN	55	P	III	I	0,5	ER
SMF	71	P	III	I	0,7	ER
MCZ	74	P	III	I	0,5	ER
TMC	60	P	I	I	0,6	ER
MFBR	52	P	III	I	0,7	ER
EOM	56	P	IV	H	0,5	ER
MAJJ	56	P	III	I	0,5	ER
SGF	27	P	I	I	0,5	ER
SMRL	56	P	III	I	0,5	ER
DGS	71	P	III	I	0,5	ER
HMBR	24	P	I	I	0,5	ER
A M S	48	P	IV	H	0,7	ER
CFB	47	P	III	I	0,5	ER
MCV	31	P	I	I	0,6	ER
TLO	58	P	I	I	0,5	ER
ECLH	40	P	I	I	3,6	ER
EAEA	34	P	I	L	7,6	ER
MJSP	22	P	I	I	1,4	ER
DEC	36	P	I	H	1,3	ER
LMJS	58	P	IV	H	0,6	ER
EPB	76	P	I	L	0,5	ER
MSLP	54	P	I	L	0,7	ER
ESS	49	P	III	I	6,3	ER
CRAR	53	P	I	L	7,1	ER
ALGCO	30	P	I	L	0,5	ER
JCBC	36	P	I	I	0,5	ER
SSR	23	P	I	I	6,2	ER
MGBS	46	P	I	I	1,6	ER
TNP	44	P	I	I	17,3	ER
CAES	40	P	I	L	1,1	ER
ACTS	22	F	I	I	81	IncR
MLLMS	35	F	III	I	0,9	IncR
JJS	46	F	III	I	3,9	IncR
RA	39	P	I	I	0,5	IncR
JNJ	63	P	IV	H	0,6	IncR
LHV	61	P	IV	H	15,7	IncR
MCSPS	55	P	III	I	40,4	IncR
MSS	27	P	I	H	47	IncR
EFR	45	P	I	L	6,3	IncR
RNS	29	P	I	I	20,7	IncR

F: Follicular carcinoma, P: Papillary carcinoma, I: Indermediate risk, H: High risk, L: Low risk, sTg: Stimulated Tg, IndR: Indeterminate response, ER Excellent response, IncR: Incomplete response.
